# ICAM-5 affects spine maturation by regulation of NMDA receptor binding to α-actinin

**DOI:** 10.1242/bio.201410439

**Published:** 2015-01-08

**Authors:** Lin Ning, Sonja Paetau, Henrietta Nyman-Huttunen, Li Tian, Carl G. Gahmberg

**Affiliations:** 1 Division of Biochemistry and Biotechnology, Faculty of Biological and Environmental Sciences, University of Helsinki, Viikinkaari 5, FIN-00014, Helsinki, Finland; 2 Neuroscience Center, University of Helsinki, Viikinkaari 4, FIN-00014, Helsinki, Finland

**Keywords:** ICAM-5, Integrin, Actinin, Cell adhesion, Spine maturation

## Abstract

ICAM-5 is a negative regulator of dendritic spine maturation and facilitates the formation of filopodia. Its absence results in improved memory functions, but the mechanisms have remained poorly understood. Activation of NMDA receptors induces ICAM-5 ectodomain cleavage through a matrix metalloproteinase (MMP)-dependent pathway, which promotes spine maturation and synapse formation. Here, we report a novel, ICAM-5-dependent mechanism underlying spine maturation by regulating the dynamics and synaptic distribution of α-actinin. We found that GluN1 and ICAM-5 partially compete for the binding to α-actinin; deletion of the cytoplasmic tail of ICAM-5 or ablation of the gene resulted in increased association of GluN1 with α-actinin, whereas internalization of ICAM-5 peptide perturbed the GluN1/α-actinin interaction. NMDA treatment decreased α-actinin binding to ICAM-5, and increased the binding to GluN1. Proper synaptic distribution of α-actinin requires the ICAM-5 cytoplasmic domain, without which α-actinin tended to accumulate in filopodia, leading to F-actin reorganization. The results indicate that ICAM-5 retards spine maturation by preventing reorganization of the actin cytoskeleton, but NMDA receptor activation is sufficient to relieve the brake and promote the maturation of spines.

## INTRODUCTION

In the central nervous system, dendritic spines, the post-synaptic components of excitatory synapses, are small protrusions arising from the dendritic shafts. It is generally agreed that the flexible, filamentous nascent spines eventually turn into stable, mushroom-shaped spines as synapses mature. Modification of spine morphology is directly driven by polymerization and depolymerization of actin and a large number of proteins have been implicated in the regulation of actin reorganization underlying spine maturation (Ethell and Pasquale, 2005; Hotulainen and Hoogenraad, 2010). Abnormalities in spines are intimately associated with a multitude of neurological disorders.

The intercellular cell adhesion molecule-5 (ICAM-5, telencephalin) is a dendrite-specific adhesion molecule with nine extracellular immunoglobulin (Ig)-like domains, a transmembrane segment and a cytoplasmic domain (Yoshihara et al., 1994; Gahmberg, 1997). ICAM-5 regulates both immune response and neuronal development in the brain (Gahmberg et al., 2008; Tian et al., 2008). The membrane-bound, full length ICAM-5 serves as a negative regulator of spine maturation and synapse formation (Yoshihara et al., 2009). β1 integrins from the pre-synaptic terminals, one of the counter receptors for ICAM-5, bind to ICAM-5 preventing further spine maturation (Ning et al., 2013). Ablation of ICAM-5 gene leads to accelerated filopodia-to-spine transition, strengthened pre- and post-synaptic contacts and increased frequency of miniature excitatory postsynaptic currents (mEPSC) in cultured neurons (Matsuno et al., 2006; Ning et al., 2013). ICAM-5-deficient mice showed improved hippocampus-related learning and memory, enhanced hippocampal long-term potentiation (LTP) and promoted synaptic plasticity during a critical period of visual cortex formation (Nakamura et al., 2001; Barkat et al., 2011).

α-Actinin is an actin binding protein, which forms anti-parallel homodimers and hinges actin filaments into bundles (Sjöblom et al., 2008). In neurons, α-actinin is concentrated in spine heads (Wyszynski et al., 1998). Overexpression of α-actinin-2 in cultured neurons led to alteration in the length and size of spines and synaptic protein recruitment (Nakagawa et al., 2004; Hoe et al., 2009).

A number of membrane proteins are anchored to the actin cytoskeleton through binding to α-actinin (Otey et al., 1990; Carpén et al., 1992; Wyszynski et al., 1997; Burgueño et al., 2003; Nyman-Huttunen et al., 2006). NMDA receptors, a subtype of glutamate receptors in excitatory synapses, colocalize with α-actinin in mature spine heads. They bind to α-actinin via the cytoplasmic domains of the GluN1 and GluN2 subunits (Wyszynski et al., 1997; Wyszynski et al., 1998; Dunah et al., 2000). Importantly, the cytoplasmic domain of ICAM-5 also binds to α-actinin (Nyman-Huttunen et al., 2006).

Previously, it was shown that activation of NMDA receptor induced an MMP-dependent cleavage of the ICAM-5 ectodomain, which promoted spine maturation, accompanied by LTP initiation (Tian et al., 2007; Conant et al., 2010). This cleavage resulted in dissociation of the ICAM-5 cytoplasmic tail from the actin cytoskeleton, indicating a role for the cytoplasmic domain during spine maturation (Tian et al., 2007). However, it has remained unclear how the cytoplasmic tail of ICAM-5 affects the actin cytoskeleton reorganization in spine morphogenesis.

Therefore, we proceeded to study the interplay between ICAM-5, NMDA receptors and α-actinin, in response to neuronal activity. We found that ICAM-5 dissociates from α-actinin upon activation of NMDA receptors, followed by an increase in GluN1 binding to α-actinin resulting in maturation of dendritic spines.

## RESULTS

### Localization of α-actinin is developmentally regulated

**Fig. 1.**
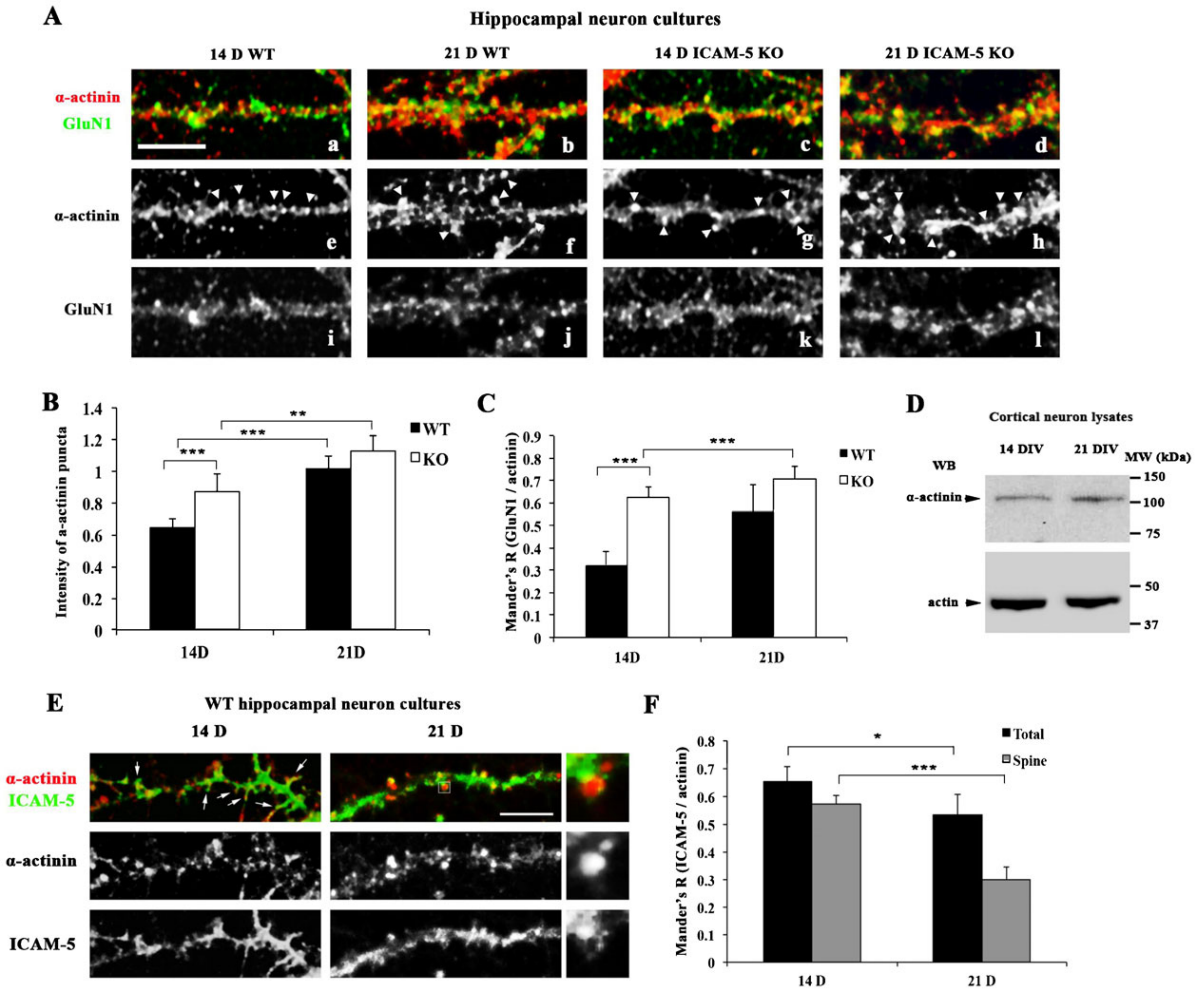
Localization of GluN1/ICAM-5/α-actinin during spine maturation. (A) Cultured hippocampal neurons were fixed at 14 and 21 DIV and immunostained for GluN1 (green) and α-actinin (red). α-Actinin exhibited a punctuated expression pattern, indicated by arrowheads (e, f, g, and h). At 14 DIV, ICAM-5 KO neurons contain larger and brighter α-actinin punctae in comparison with WT (g vs e). With maturation, α-actinin intensity increased in both WT and ICAM-5 KO neurons (f and h). Colocalization between GluN1 and α-actinin was higher in KO neurons at 14 DIV, while the difference became insignificant at 21 DIV (yellow area, a, b, c, and d). Scale bar = 10 µm. (B) α-Actinin puncta ROIs were selected manually after background subtraction. The total fluorescent intensity within the ROIs was analyzed for each group of neurons. Integrated fluorescent intensity reflects both the size and brightness of the punctae. (C) Colocalization between GluN1 and α-actinin was analyzed among all neuron groups. The level of colocalization was measured by Manders' correlation coefficient (Manders' R). (B) and (C) data were analyzed from three independent experiments. More than 500 spines were analyzed for each genotype. **p<0.01; ***p<0.001. (D) 17 DIV cortical neurons were lysed and 5 µg cell lysates were analyzed by SDS-PAGE followed by western blotting. Expression of α-actinin slightly increased from 14–21 DIV. Actin blots show that similar amounts of lysate were loaded. (E) Cultured hippocampal neurons were fixed at 14 and 21 DIV and immunostained for ICAM-5 (green) and α-actinin (red). In 14 DIV cultures, α-actinin was mostly expressed along dendritic shafts where it colocalized with ICAM-5. A small population of α-actinin was also detected in filopodia (arrows). In 21 DIV neurons, α-actinin was concentrated in spine heads. ICAM-5 was excluded from some spine heads and only partially colocalized with α-actinin. Scale bar = 10 µm. Enlarged view: α-actinin is concentrated in a spine head while ICAM-5 is expressed in the neck of the spine. (F) Colocalization between ICAM-5 and α-actinin was analyzed in the whole dendrites and in spines. *p<0.05, ***p<0.001.

To study the correlation of ICAM-5, GluN1 and α-actinin in spine maturation, we examined the distribution patterns of the proteins and the colocalization of them during development using immunofluorescent staining. Hippocampal neurons were cultured until 14–21 day in vitro (DIV), corresponding to the time when the majority of spines undergo morphological and functional maturation. After fixation, neurons were co-stained for GluN1 and α-actinin. The specificity of staining antibodies was shown in previous studies (Wyszynski et al., 1998). For staining involving GluN1, methanol fixation was performed to expose the epitopes to the primary antibody. Both GluN1 and α-actinin exhibited a punctuated pattern (Fig. 1A), as reported earlier (Rao and Craig, 1997; Wyszynski et al., 1998; Hodges et al., 2014). However, it was difficult to visualize the morphology of spines due to the methanol fixation, which quenches fluorescent proteins and denatures actin. To better show the localization of the α-actinin punctae, we co-stained PFA-fixed neurons with α-actinin, F-actin, PSD-95, and synapsin I, respectively. α-Actinin largely colocalizes with actin. Particularly, α-actinin punctae almost overlapped with the actin-rich area along dendritic shafts, suggesting that α-actinin punctae mostly are located in spines. In addition, PSD-95 and synapsin I both colocalize with α-actinin. (supplementary material Fig. S1). Therefore, we consider α-actinin puncta partially representing mature spines. An increase in the size and intensity of α-actinin punctae was observed when comparing neurons at 21 DIV (Fig. 1A,b,f, arrowheads, Fig. 1B) with 14 DIV neurons (Fig. 1A,a,e, arrowheads, Fig. 1B). Interestingly, at 14 DIV, a similar increase of α-actinin puncta size was also seen in the ICAM-5 KO neurons in comparison with WT cultures (Fig. 1A,c,g). This is consistent with the previous finding that ICAM-5 KO neurons express more mature spines in developing neurons (Matsuno et al., 2006). At 21 DIV, the difference in puncta size was less significant between WT and ICAM-5 KO neurons (Fig. 1A,f,h,B).

The colocalization of GluN1 and α-actinin also changes during development. In WT neurons, GluN1 and α-actinin colocalization increased two fold from 14 to 21 DIV (Fig. 1A,a,b,C). At 14 DIV, the colocalization was significantly higher in ICAM-5 KO neurons than that of WT (Fig. 1 A,a,c,C); while at 21 DIV, the difference became insignificant (Fig. 1A,b,d,C). The increased colocalization is largely due to redistribution of α-actinin, since there was only a slight increase of the overall α-actinin expression from 14 to 21 DIV (Fig. 1D).

Colocalization of ICAM-5 and α-actinin was also examined. Cultured hippocampal neurons were fixed at 14 and 21 DIV and double stained for ICAM-5 (Fig. 1E, green) and α-actinin (Fig. 1E, red). ICAM-5 immunoreactivity is abundant in filopodia and immature spines, but weaker in mature spines (Matsuno et al., 2006; Tian et al., 2007). At 14 DIV, α-actinin puncta colocalized with ICAM-5 along the shafts as well as in filopodia and spines (Fig. 1E, arrows). At 21 DIV, α-actinin immunoreactivity in dendritic shafts became weaker and more diffuse and the colocalization with ICAM-5 decreased in shafts as well as in spines (Fig. 1E,F). However α-actinin was highly concentrated in puncta adjacent to dendritic shafts. Interestingly, it was often observed that in α-actinin clusters, ICAM-5 immunoreactivity was excluded (Fig. 1E,F).

These results suggest that the interaction of ICAM-5 with α-actinin is reciprocally correlated with that of GluN1 and α-actinin. ICAM-5/α-actinin colocalization was more obvious in young spines, before α-actinin became enriched in mature spine heads.

### ICAM-5 and GluN1 have overlapping binding region in α-actinin

**Fig. 2.**
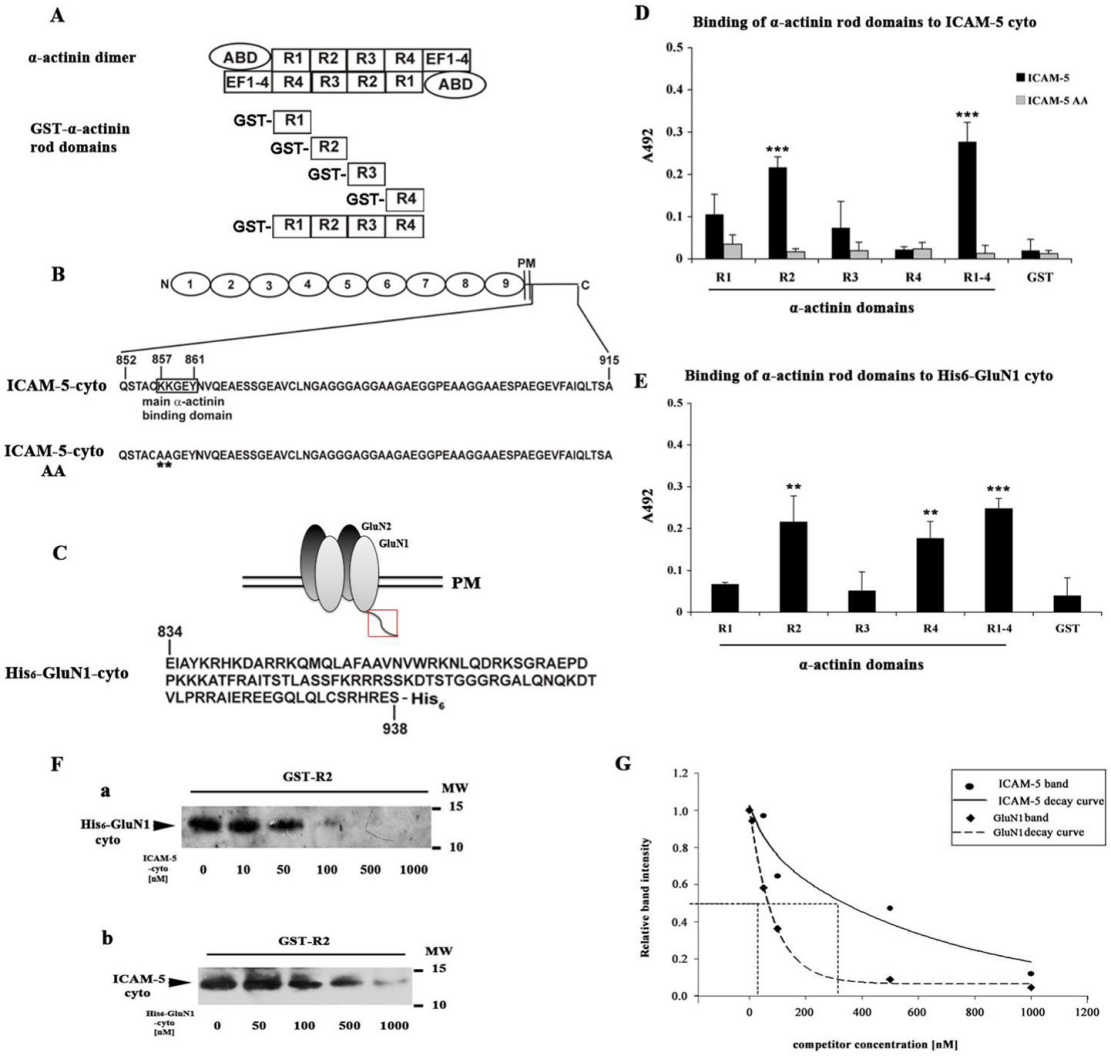
Competition between ICAM-5 and GluN1 in binding to α-actinin. Schematic structures of proteins used in ELISA. (A) α-Actinin homodimers and recombinant GST- α-actinin R1-R4 representing the whole rod domains, (B) Full length ICAM-5 and the amino acid sequence of the ICAM-5 cytoplasmic domain. The sequence KKGEY 857–861 binds to α-actinin (Nyman-Huttunen et al., 2006). The WT cytoplasmic tail of ICAM-5 and a mutant with two lysines mutated to alanine (ICAM-5 AA) were used in the assays. (C) An NMDAR tetramer containing two GluN1 and two GluN2 subunits. The amino acid sequence of the GluN1 cytoplasmic domain (red frame) is shown. A hexahistidine tag was added to its C-terminal. (D) Binding of the ICAM-5 cytoplasmic domain or the ICAM-5 AA mutant to α-actinin R domains. (E) Binding of the His_6_-GluN1 cytoplasmic domain to α-actinin R domains. All values were normalized to the level BSA binding. **p<0.01, ***p<0.001. (F) Two µg of purified GST-α-actinin-R2 was immobilized to glutathione-sepharose, and incubated with purified His_6_-GluN1 (a) or ICAM-5 (b) cytoplasmic proteins, respectively, with increasing molar amounts of ICAM-5 (a) or His_6_-GluN1 cytoplasmic domain (b). His_6_-GluN1 and ICAM-5 bound to R2 were detected by western blotting. (G) The band intensities of His_6_-GluN1 (F,a) or ICAM-5cyto (F,b) were quantitatively analyzed and plotted against the concentration of competing ICAM-5 (F,a) or His_6_-GluN1 (F,b). Data points were fitted into exponential curves for ICAM-5 and GluN1 respectively. Half maximum inhibition was calculated from the curves.

The reciprocal colocalization of ICAM-5 and GluN1 with α-actinin led us to study whether a competition between ICAM-5 and GluN1 for α-actinin binding exists. The α-actinin monomer is composed of an actin-binding domain at the NH_2_-terminal, a central rod-shaped domain containing four spectrin repeats, and a COOH-terminal EF hand domain (Sjöblom et al., 2008) (Fig. 2A,a). Our previous work showed that the peptide 857–861 (KKGEY) of ICAM-5 directly binds to the rod domain of α-actinin (Fig. 2B). The GluN1 cytoplasmic domain (Fig. 2C) also binds to α-actinin and the binding region is located in the α-actinin rod domain.

α-Actinin rod domains (Fig. 2A), WT or KK/AA mutated (Fig. 2B, ICAM-5-cyto AA) ICAM-5 cytoplasmic domains were expressed in E. coli as GST-fusion proteins and the GluN1 cytoplasmic domain as a His-tag fusion protein (Fig. 2C, His_6_-GluN1cyto). The GST tag was removed from ICAM-5 WT and the mutated protein. ICAM-5 cytoplasmic domains or His_6_-GluN1cytoplasmic domain were coated on plates and incubated with different α-actinin rod domains. As reported earlier, the full rod domain R1–R4 bound to both ICAM-5 and GluN1 cytoplasmic peptides, but not the ICAM-5 KK/AA mutant. Among four spectrin domains, the R2 domain showed the best binding to both peptides (Fig. 2D,E). In addition, the R4 domain also bound to GluN1, but not to ICAM-5 (Fig. 2E).

### Competition between ICAM-5 and GluN1 in binding to α-actinin

Since ICAM-5 and GluN1 both bind to the R2 domain of α-actinin, it was important to study whether the two proteins compete with each other for binding. Two µg of the GST-R2 fusion protein was immobilized to glutathione- sepharose. One ml of 250 nM purified His_6_-GluN1 cytoplasmic domain solution was incubated with R2-coupled sepharose in the presence of increasing amounts of the ICAM-5 cytoplasmic domain. At low concentrations of ICAM-5, GluN1 was seen to bind to R2. When the concentration of the ICAM-5 cytoplasmic domain increased, the binding of GluN1 to R2 decreased (Fig. 2F,a). Reciprocally, the His_6_-GluN1 cytoplasmic domain competed with ICAM-5 cytoplasmic domain binding (Fig. 2F,b). The half maximum inhibition of ICAM-5 to R2 required approximately 340 nM of GluN1, whereas that of GluN1 to R2 occurred at 55 nM of ICAM-5 (Fig. 2G). These results show that ICAM-5 and GluN1 compete for binding to the R2 domain of α-actinin and ICAM-5 has a somewhat higher affinity for the R2 domain.

### ICAM-5 out-competes GluN1 in binding to α-actinin *in vivo*

**Fig. 3.**
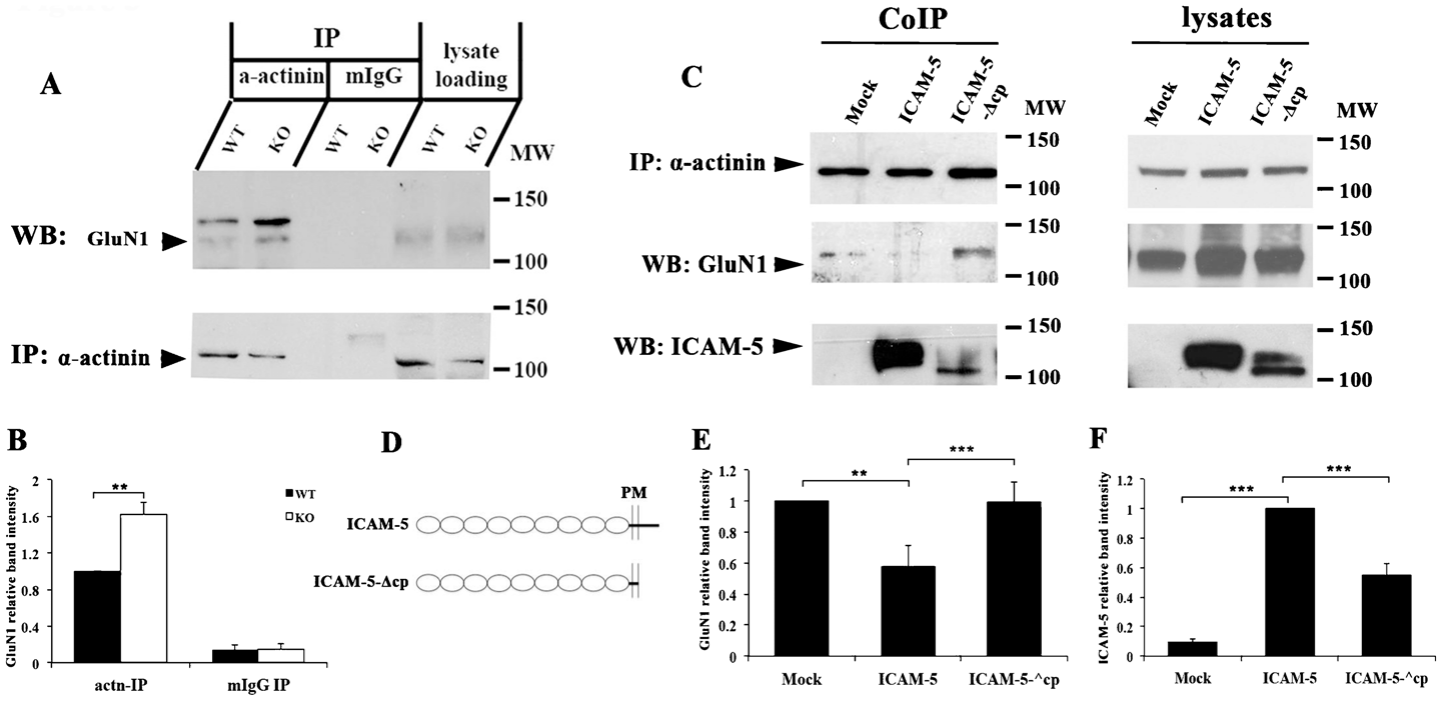
ICAM-5 interferes with the binding between GluN1 and α-actinin. (A) Two-hundred µg of brain homogenates from WT and ICAM-5 KO mice were immunoprecipitated with α-actinin antibody. The amount of GluN1 bound to α-actinin was detected by western blotting. The bands above the indicated GluN1 bands may be GluN1 in complex with other molecules. The reason that they are not seen in the lysates may be due to low concentration. (B) Relative intensity of the GluN1 bands was quantitative analyzed. **p<0.01. The experiments were repeated three times with similar results. (C) Lysates of Paju cells, transfected with empty plasmid (Mock), ICAM-5 or ICAM-5-Δcp constructs, were used for immunoprecipitation of α-actinin. The amounts of co-immunoprecipitated ICAM-5 and GluN1 was detected by western blotting (C, left panels). The constructs are schematically shown. ICAM-5-Δcp is a truncated form of the full-length ICAM-5 with the cytoplasmic domain deleted (D). Five µg lysates from IP sample was loaded for SDS-PAGE. The amount of α-actinin, ICAM-5 and GluN1 were detected by western blotting (C, right panels). Relative intensity of the GluN1 (E) and ICAM-5 (F) CoIP bands were quantitative analyzed. **p<0.01, ***p<0,001. The experiments were repeated three times with similar results.

The competition between ICAM-5 and GluN1 in binding to α-actinin led us to study whether the interaction of α-actinin and GluN1 is altered by ICAM-5 *in vivo*. Postnatal day 14 (P14) WT and ICAM-5 KO mouse brains were used. When α-actinin was immunoprecipitated, the amount of co-immunoprecipitated GluN1 was higher from ICAM-5 KO mouse brain as compared to that of WT mice (Fig. 3A,B). Lysate loading control showed that the overall expression of GluN1 and α-actinin was similar in both WT and ICAM-5 KO brains. To further study the interference of the ICAM-5 cytoplasmic tail in GluN1/α-actinin binding, we took advantage of the neuronal cell line Paju expressing full-length ICAM-5 (Fig. 3C,D, ICAM-5), cytoplasmic tail-deleted ICAM-5 (Fig. 3C,D, ICAM-5-Δcp), or cells transfected with an empty plasmid (Fig. 3C, Mock). GFP-GluN1 was transiently transfected into Paju cells and α-actinin was immunoprecipitated from the cell lysates. In mock or ICAM-5-Δcp cells, the binding between GluN1 and α-actinin increased as compared to cells expressing full-length ICAM-5 (Fig. 3C,E). The amount of ICAM-5 bound to α-actinin greatly decreased when lacking of the cytoplasmic domain (Fig. 3C,F). This means that the cytoplasmic tail of ICAM-5 interfered with GluN1 binding to α-actinin.

### The cytoplasmic domain of ICAM-5 is required to prevent α-actinin clustering

**Fig. 4.**
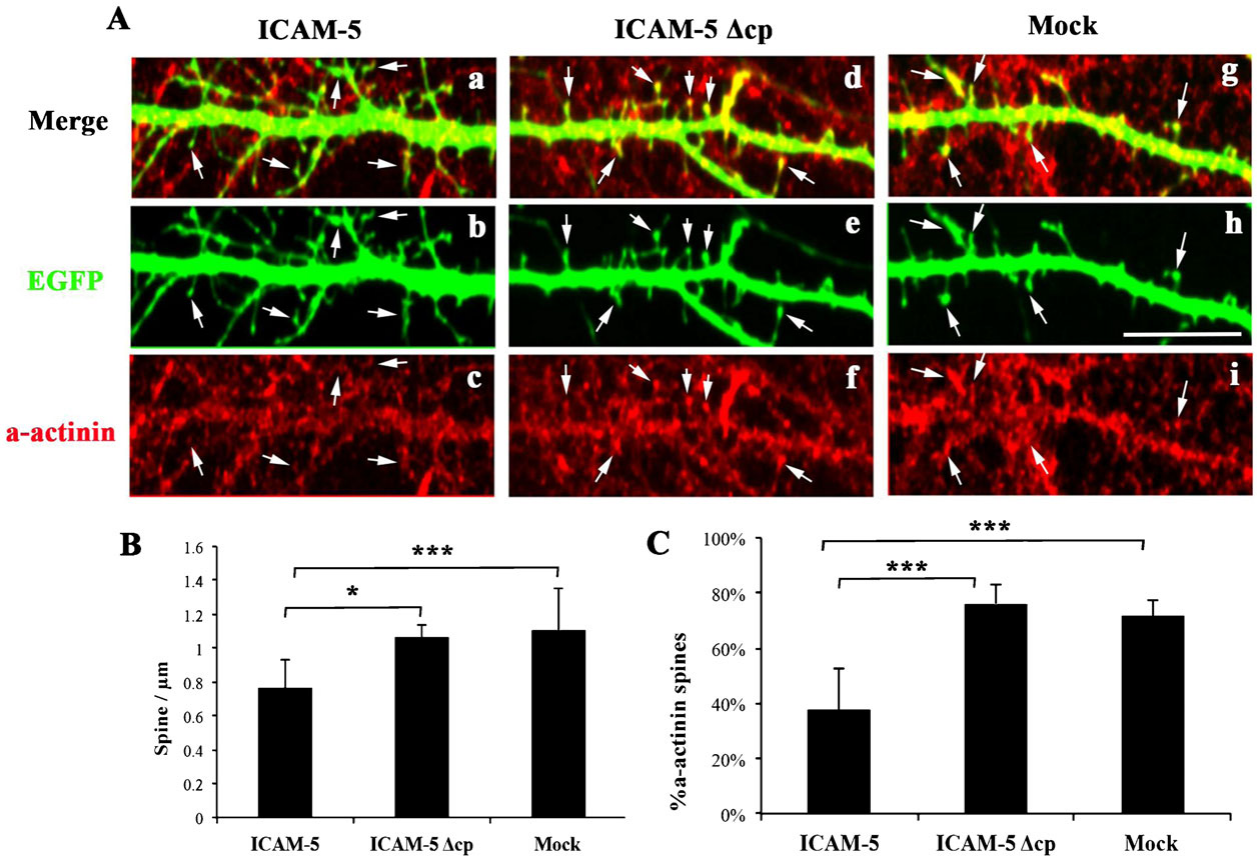
ICAM-5 rescue of spine morphology and α-actinin distribution. (A) 11 DIV hippocampal neurons were co-transfected with EGFP and ICAM-5 constructs (PEF-BOS-ICAM-5, PEF-BOS-ICAM-5 Δcp or empty PEF-BOS plasmid) at 1: 10 ratio, and fixed at 12 DIV. Cells were stained for α-actinin and the fine structure of dendrites visualized by EGFP. Arrows indicate spines. Scale bar = 10 µm. The number of mature spines (B), and the amount of α-actinin present in spine heads (C) was quantified. Data were analyzed from three independent experiments. Analyzed spine number: ICAM-5, 459; ICAM-5 Δcp, 639; Mock, 663.

Since the ICAM-5 cytoplasmic domain perturbs GluN1 and α-actinin binding, it was intriguing to study its effect on α-actinin distribution. 11 DIV ICAM-5 KO neurons were co-transfected with EGFP and ICAM-5 constructs with (Fig. 4A, ICAM-5) or without (Fig. 4A, ICAM-5 Δcp) the cytoplasmic domain, or an empty vector (Fig. 4A, Mock). The amounts of ICAM-5 constructs were excessive relative to EGFP, and 97% of EGFP-transfected cells were ICAM-5 positive (data not shown). Compared to mock and ICAM-5 Δcp cells (Fig. 4A), the ICAM-5-transfected neurons (Fig. 4A,b) contained more immature spines, with longer spine necks and smaller spine heads (Fig. 4B), suggesting that the cytoplasmic domain of ICAM-5 is required to delay spine maturation.

In addition, in mock and ICAM-5-Δcp transfected neurons, α-actinin immunoreactivity formed clusters, which often located within spine heads (Fig. 4A,f,i,d,g, arrows); while full-length ICAM-5 transfection was able to prevent the clustering of α-actinin (Fig. 4A,a,c). This result indicates a role of the ICAM-5 cytoplasmic domain in regulating α-actinin translocation in spine heads.

### Activation of NMDA receptors alters the interaction of ICAM-5 and GluN1 with α-actinin

Previous studies have shown that NMDAR-dependent spine maturation is triggered by ICAM-5 ectodomain cleavage followed by detachment of its cytoplasmic tail from the actin cytoskeleton (Tian et al., 2007). The mechanism has remained incompletely understood.

**Fig. 5.**
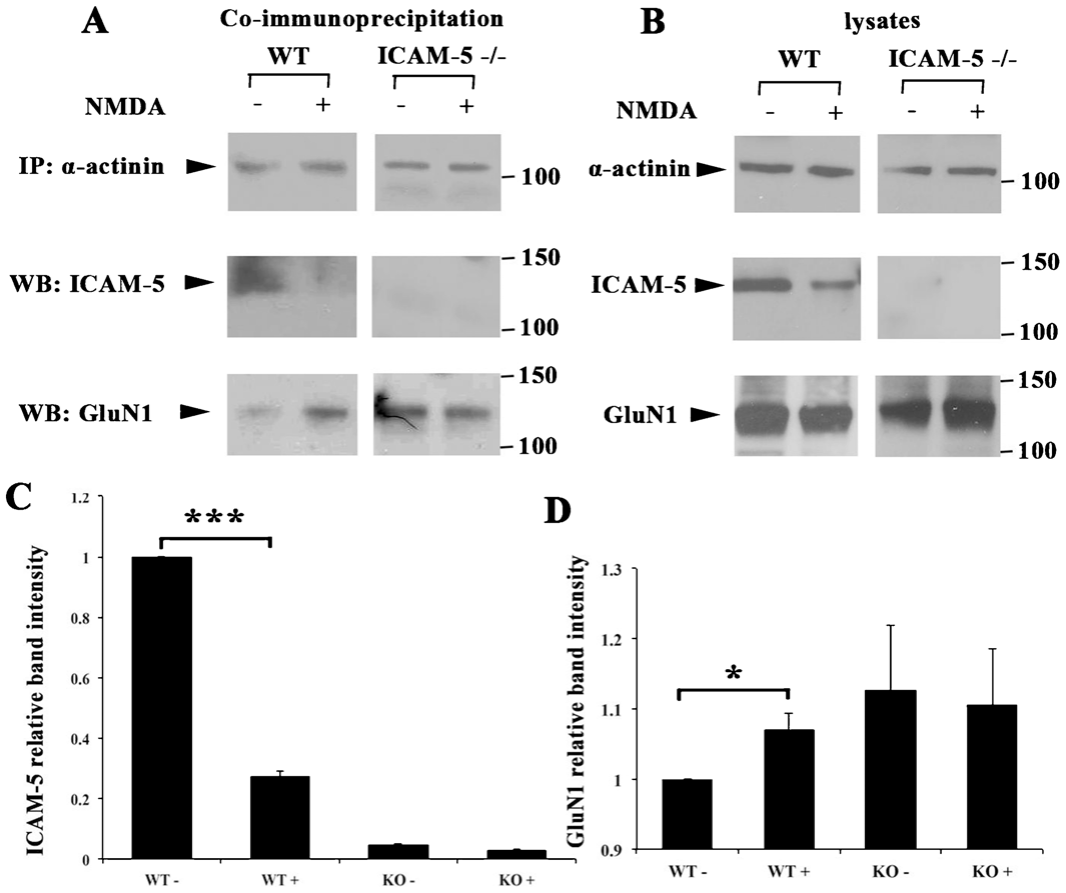
NMDA stimulation results in changes in ICAM-5/α-actinin/GluN1 binding. (A) 13 DIV cultured WT and ICAM-5 KO cortical neurons were left untreated or treated with 20 µM NMDA for 1 h. Cell lysates were immunoprecipitated with α-actinin antibodies. The amount of bound ICAM-5 and GluN1 was detected by western blotting. (B) Five µg lysates from IP sample was loaded for SDS-PAGE. The amount of α-actinin, ICAM-5 and GluN1 were detected by western blotting. Relative intensities of ICAM-5 (C) and GluN1 (D) bands were quantitated. The same experiment was repeated three times with similar results. *p<0.05, ***p<0.001.

We first examined whether NMDAR activation causes ICAM-5 dissociation from α-actinin. 13 DIV cultured cortical neurons from WT and ICAM-5 −/− mice were treated with 20 µM NMDA for 1 h and α-actinin was immunoprecipitated. In WT neurons, the amount of ICAM-5 bound to α-actinin decreased after NMDA treatment (Fig. 5A,C), and, concomitantly, bound GluN1 increased (Fig. 5A,D). Lysate loading showed that the amounts of GluN1 and α-actinin stayed about the same after NMDA treatment, while ICAM-5 level decreased due to cleavage (Fig. 5B). However, the decrease of ICAM-5 binding to α-actinin was not due to a total loss of ICAM-5. We found that there was ICAM-5 remaining in the unbound fraction (data not shown), suggesting that the amount of ICAM-5 in the lysate exceeded the binding capacity of α-actinin. However, in ICAM-5 −/− neurons, NMDA treatment failed to change the binding of GluN1 and α-actinin significantly (Fig. 5A,D). The results show that NMDA receptor activation induced ICAM-5 dissociation from α-actinin followed by increased GluN1 binding to α-actinin.

**Fig. 6.**
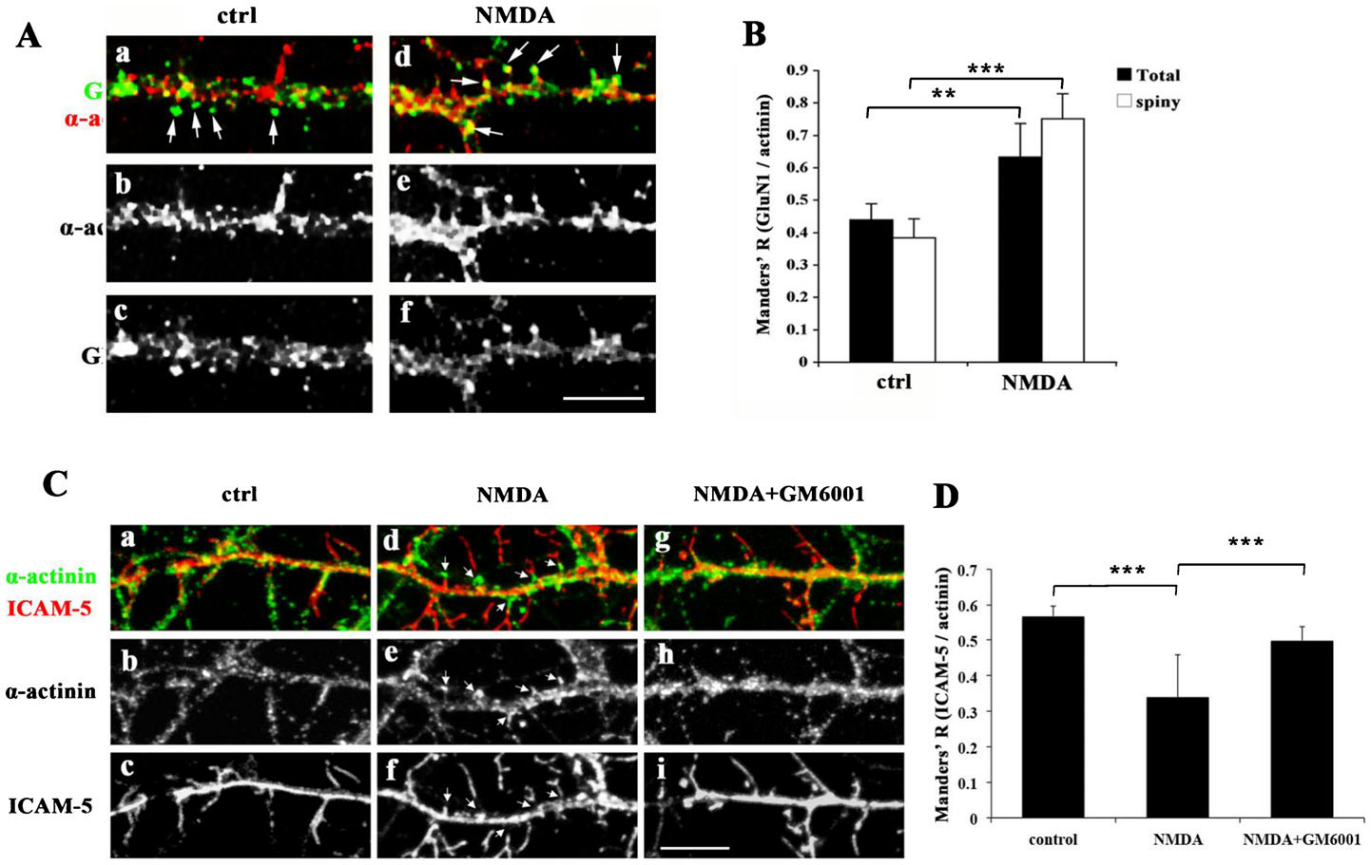
NMDA stimulation results in changes in ICAM-5/α-actinin/GluN1 colocalization. (A) 12 DIV cultured hippocampal neurons were left untreated, or treated with 20 µM NMDA for 1 h. After fixation, cells were immunostained for GluN1 (green) and α-actinin (red). α-Actinin immunoreactivity is clustered in puncta, where the colocalization of α-actinin and GluN1 was increased (arrows). Scale bar = 10 µm. (B) Quantitative analysis of GluN1/α-actinin colocalization. Data were analyzed from three independent experiments. Control, n = 15 neurons; NMDA, n = 17 neurons. The number of analyzed actinin puncta: control, 637; NMDA, 612. **p<0.01, ***p<0.001. (C) 12 DIV cultured neurons were left untreated (ctrl) or treated with 20 µM NMDA or NMDA together with 20 µM GM6001 (NMDA+GM6001), for 1 h. After fixation, cells were immunostained for ICAM-5 (red) and α-actinin (green). Arrows indicate the α-actinin clusters after NMDA treatment (d–f). Scale bar = 10 µm. The colocalization between α-actinin and ICAM-5 (D) was quantitatively analyzed. Data was analyzed from three independent experiments. Control, n = 15 neurons; +NMDA, n = 19 neurons, NMDA+GM6001, n = 19. At least 500 actin clusters were analyzed in each case. *p<0.05, **p<0.01.

The effects of NMDA treatment on colocalization of GluN1/α-actinin and ICAM-5/α-actinin were studied by immunofluorescent staining. The colocalization between GluN1 and α-actinin increased after NMDA treatment, and the increase occurred both along the shafts as well as in spines (Fig. 6A,B). Concomitantly, a significant decrease of colocalization between α-actinin and ICAM-5 was found after NMDA treatment (Fig. 6C,a vs d, Fig. 6D). The colocalization results are consistent with the co-immunoprecipitation results (Fig. 5). Notably, increased colocalization was often found in α-actinin puncta (Fig. 6A,a vs d, arrows). Interestingly, these clusters were often seen on the tips or at the roots of filopodia, which were largely devoid of ICAM-5 immunoreactivity (Fig. 6C,d–f, arrows). Moreover, the MMP inhibitor GM6001 reversed the effect of NMDA treatment on α-actinin clustering as well as colocalization of ICAM-5/α-actinin (Fig. 6C,g–i,D).

### Time-lapse imaging of α-actinin

**Fig. 7.**
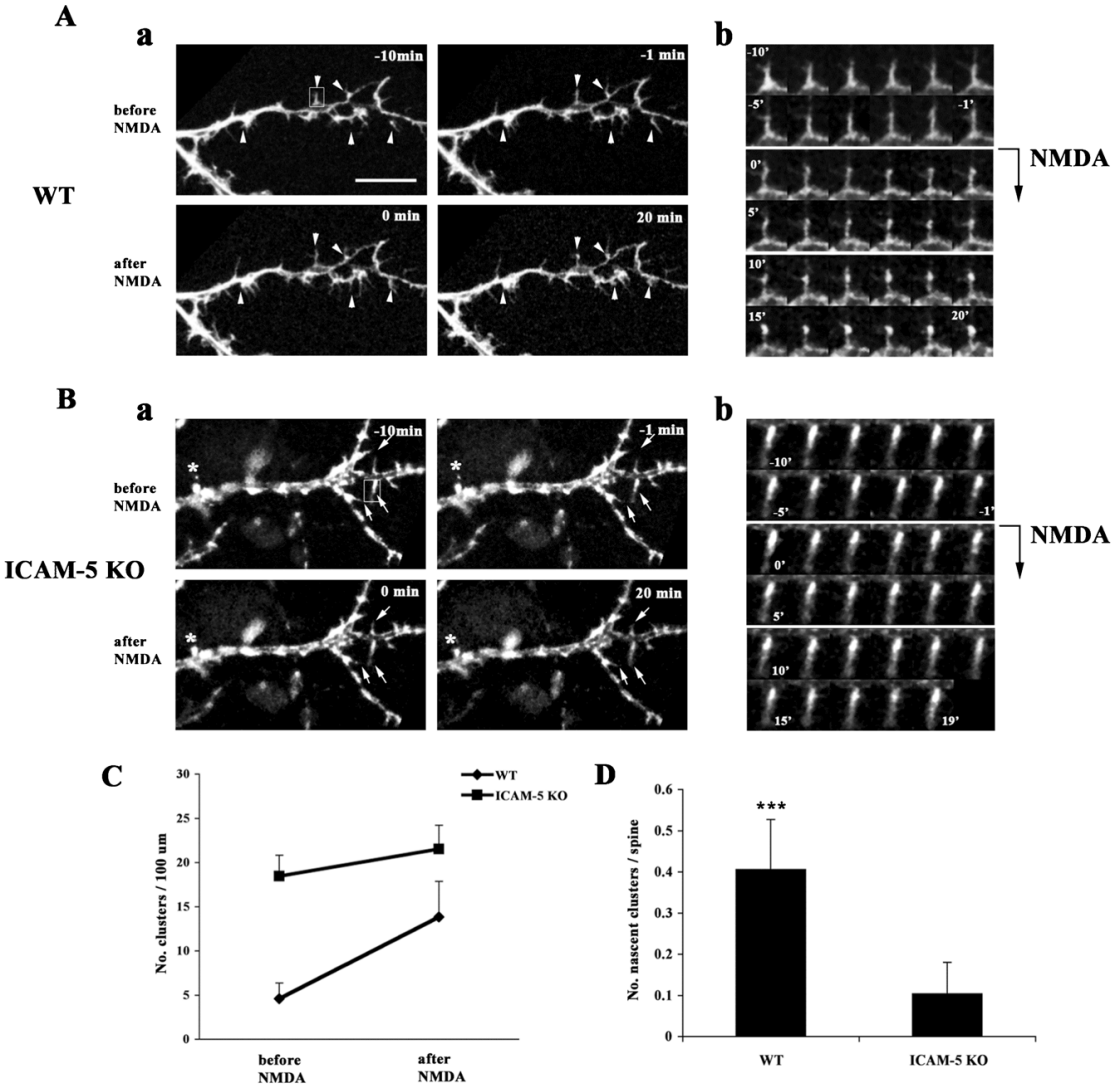
NMDA stimulation facilitates clustering of α-actinin. 11 DIV hippocampal neurons from WT (A) or ICAM-5 KO (B) mice were transfected with mKATE-α-actinin. Neurons were imaged 10 min before, and 20 min after treatment of 20 µM NMDA. (A) In WT neurons, α-actinin formed clusters at the tips or the roots of filopodia after NMDA treatment (arrowheads). (B) In ICAM-5 KO neurons, α-actinin failed to show significant clustering in response to NMDA (arrows). Scale bar = 10 µm. Framed filopodia are shown in an enlarged view (A,b and B,b). (C) Quantitative analysis of α-actinin clusters before and after NMDA treatment in WT and ICAM-5 KO neurons. (D) Quantitative analysis of the number of nascent α-actinin clusters located in spines/filopodia in response to NMDA treatment. Data were obtained from three independent experiments. WT, n = 14 neurons; ICAM-5 −/−, n = 11 neurons. ***p<0.001.

To further study the clustering of α-actinin, we monitored the dynamics of α-actinin by time-lapse imaging. Cultured hippocampal neurons were transfected with mKATE (a far-red fluorescent protein)-α-actinin at 11 DIV and imaged at 12 DIV. We confirmed by immunostaining that the transfected plasmid as compared to the endogenous α-actinin was not overexpressed by the time of imaging. We focused on a time frame of 20 min after NMDA treatment (Conant et al., 2010). In WT neurons, α-actinin was evenly distributed along the shafts, weakly expressed in filopodia and concentrated in a small number of clusters. In filopodia α-actinin was highly motile, moving randomly in a zigzag manner along the length of filopodia (Fig. 7A,a, before NMDA; supplementary material Movie 1). Upon NMDA treatment, rapid clustering of α-actinin occurred. After 20 min of NMDA treatment, the number of clusters increased two fold (Fig. 7C; supplementary material Movie 2). There were two sorts of α-actinin movement in filopodia: (1) α-actinin moved toward the tips of filopodia where it formed clusters (Fig. 7A,a, framed filopodia, Fig. 7A,b); (2) α-actinin retracted, moved toward dendritic shafts and gradually formed clusters at the roots of filopodia adjacent to the shafts (Fig. 7A,a, arrowheads). The different directions of α-actinin movements at least partially contributed to the variability of spine morphology. The former one will likely facilitate the formation of thin spines whereas the latter one possibly results in the formation of stubby spines or mushroom spines.

In ICAM-5 −/− neurons, α-actinin formed clusters at 12 DIV (Fig. 7B,a, before NMDA; supplementary material Movie 3) and the number of clusters was more than two fold higher than that of WT cells (Fig. 7C). α-Actinin was concentrated in these clusters and did not show much movement. A small fraction of filopodia-like structures were observed. α-Actinin maintained its mobility in these structures (Fig. 7B,a, arrows). In contrast to WT neurons, most filopodia from ICAM-5 −/− cells showed little response upon NMDA treatment, neither showing directional movement nor forming clusters (Fig. 7B,a, arrows and Fig. 7B,b; supplementary material Movie 4). Occasionally, shrinkage of α-actinin clusters was found after NMDA treatment (Fig. 7B, asterisks). On the contrary, WT neurons formed three times more nascent clusters of α-actinin in filopodia/spines during 20 min of NMDA treatment (Fig. 7D). These results show that NMDA-induced ICAM-5 cleavage promotes the formation of α-actinin clusters.

### NMDA treatment induces co-clustering of α-actinin with F-actin

**Fig. 8.**
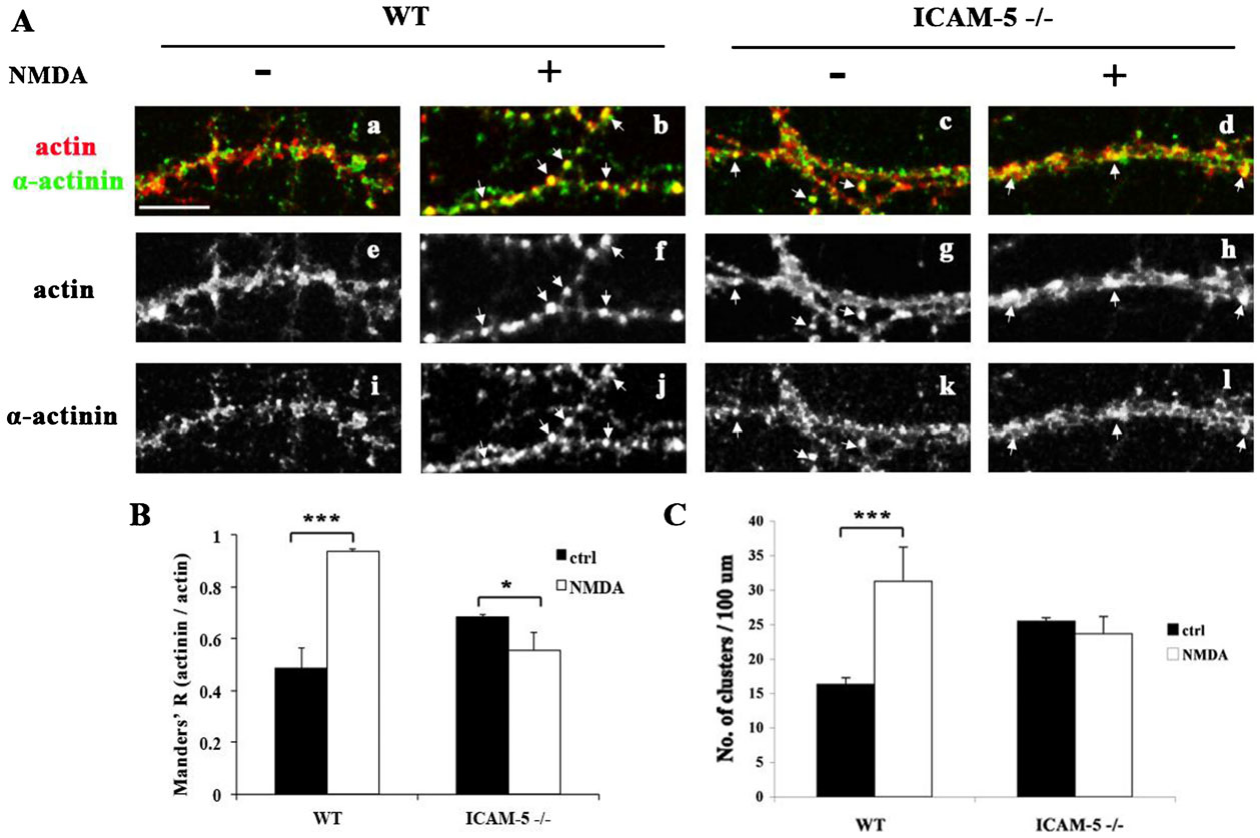
NMDA stimulation increases colocalization of α-actinin with F-actin and induces F-actin clustering. (A) 12 DIV cultured WT and ICAM-5 KO neurons were left untreated and treated with 10 µM NMDA. After fixation, the cells were stained with phalloidin (red) and for α-actinin (green). NMDA stimulation resulted in an increase in F-actin clusters and α-actinin co-clustered with F-actin (arrows) in WT neurons. These changes were not seen in ICAM-5 KO neurons. Scale bar = 10 µm. Quantitative analysis of the number (C) of actin clusters and colocalization of α-actinin and actin within actin clusters (B). WT, −NMDA, n = 12; WT, +NMDA, n = 14; ICAM-5 −/−, −NMDA, n = 13; ICAM-5 −/−, +NMDA, n = 10. At least 500 actin clusters were analyzed in each case. *p<0.05, ***p<0.001.

F-actin, the major cytoskeletal component of dendritic spines, is physically and functionally associated with α-actinin. Since NMDA treatment induced ICAM-5 dependent α-actinin clustering, it could also affect the integrity of the actin cytoskeleton. NMDA treated neurons were fixed and stained with phalloidin (Fig. 8A, red) and for α-actinin (Fig. 8A, green). Similar to α-actinin, in WT neurons, F-actin immunoreactivity became highly concentrated, and formed bright clusters along the dendritic shafts after NMDA treatment, with a significant increased number of puncta (Fig. 8A,b,f, arrows, Fig. 8C). Notably, colocalization of actin and α-actinin was also greatly increased in these actin puncta (Fig. 8A,a,b,B), suggesting co-clustering of α-actinin and F-actin. ICAM-5 KO neurons showed few changes in the number of actin puncta in response to NMDA treatment (Fig. 8A,c,g, arrows, Fig. 8C), and the colocalization of F-actin/α-actinin slightly decreased (Fig. 8A,c,d,D). These results indicate that ICAM-5 plays a regulatory role in NMDAR-activity dependent actin cytoskeleton reorganization.

## DISCUSSION

The role of ICAM-5 in spine morphology and cognitive functions, and the association between memory and actin-based spine remodeling makes it highly relevant to study the involvement of ICAM-5 in actin organization.

Spines are the cellular storage of many forms of memory, and changes in spine morphology may underlie brain plasticity and memory formation. Actin dynamics, the major driving force for shaping spine architecture, has gained research attention. Accumulating evidence pinpoint an interplay between actin organization, synaptic activity and higher brain functions (Cingolani and Goda, 2008). Electrical stimulation or pharmacological activation and inhibition of glutamate receptors resulted in altered actin dynamics and turnover time (Fischer et al., 2000; Star et al., 2002). It is also known that regulation of actin binding proteins directly affects memory and learning (Pontrello et al., 2012; Huang et al., 2013), and disruption of actin structure or inhibition of actin polymerization lead to loss of memory (Honkura et al., 2008; Krucker et al., 2000).

In this work, we have shown that removal of ICAM-5, either by gene ablation or by NMDA receptor activation, is accompanied by a switch of α-actinin binding from ICAM-5 to GluN1. Loss of interaction with ICAM-5 resulted in clustering of α-actinin in immature spines, actin cytoskeleton remodeling, and more mature spines.

A possible role of ICAM-5 and in particular its cytoplasmic domain, in filopodia and immature spines is to maintain the dynamics of α-actinin and to prevent α-actinin clustering. This interpretation is supported by the following results: (1) time-lapse imaging shows that ICAM-5 −/− neurons contain relatively stable, clustered α-actinin, whereas in WT neurons, α-actinin is highly mobile and the filopodia show increased flexibility (Nakagawa et al., 2004), (2) α-actinin tends to form puncta in neuronal areas devoid of ICAM-5 immunoreactivity, including mature spines or spines of ICAM-5 −/− neurons, (3) only ICAM-5 with an intact cytoplasmic domain was able to rescue the premature clustering of α-actinin in ICAM-5 −/− neurons, (4) activation of NMDA receptors, which decreases ICAM-5/α-actinin binding, led to α-actinin clustering and stabilization of filopodia and immature spines, ultimately giving rise to more mature spines.

Despite that the detailed mechanism remains unclear, dissociation of ICAM-5 from α-actinin is dependent on MMP-mediated ICAM-5 cleavage, since the MMP inhibitor GM6001 effectively blocked NMDA-induced ICAM-5/α-actinin de-colocalization. It is possible that when ICAM-5 loses the ectodomain, the remaining segment is prone to endocytosis and degradation. Interestingly, it is known that ICAM-5 can be removed from dendritic spines in an ADP-ribosylation factor 6 (ARF6)-dependent pathway, and then targeted for lysosomal degradation (Raemaekers et al., 2012). In fact, the remaining fragment of ICAM-5 after cleavage was barely seen suggesting degradation (supplementary material Fig. S2).

The direct reason for α-actinin clustering is still unknown. The clustering may be due to a direct physical interaction with GluN1, as a result of NMDA receptor trafficking to PSD and enrichment in spine heads during spine maturation. Another possibility is that NMDA receptor activation induces signaling pathways that regulate α-actinin dynamics. Several studies have shown that activation of glutamate receptors altered the activity or distribution of actin binding proteins, which in turn regulate actin dynamics underlying spine morphology and synaptic function (Pontrello et al., 2012; Sekino et al., 2006; Takahashi et al., 2009; Star et al., 2002).

Using live-cell imaging, we observed that upon NMDA receptor activation, α-actinin rapidly moved toward the heads or the roots of filopodia. The spines then became less mobile and enlarged in α-actinin clustered areas. Hotulainen and co-workers earlier reported that the head and the root of filopodia are the major sites for actin polymerization. During spine maturation, actin-binding proteins such as Arp2/3 facilitate actin branching, and therefore promote spine head enlargement (Hotulainen et al., 2009). Certainly, a stable actin filament network requires bundling activity by actin cross-linking proteins. It is possible that an increased local concentration of α-actinin facilitates actin filament bundling and contributes to stabilization of actin filaments resulting in more mature spines. In agreement with this, we found that F-actin co-clustered with α-actinin in response to NMDA receptor activation. The interrelationship among actin assembly, synaptic efficacy and memory functions has been extensively studied. Spine maturation includes stabilization of the actin cytoskeleton and an increased growth and complexity of the spinal actin network, resulting in increased spine volume, higher AMPAR/NMDAR ratio, and enhanced synaptic transmission capacity. Therefore, altered α-actinin dynamics and stabilization of the actin cytoskeleton in ICAM-5 −/− neurons may contribute to the improved memory and higher LTP in ICAM-5 −/− mice.

Previous studies have shown that α-actinin is involved in the regulation of NMDAR function in a Ca^2+^-dependent fashion. When NMDARs are activated, in the presence of Ca^2+^, calmodulin outcompetes α-actinin for GluN1 binding, and consequently inactivates NMDARs (Krupp et al., 1999; Leonard et al., 2002; Merrill et al., 2007). The competition between α-actinin and calmodulin is implicated as a protective mechanism that prevents neurotoxicity in mature spines due to over-excitation of NMDAR. It is apparent that activation of NMDAR could generate two different results in terms of NMDAR and α-actinin binding: (1) inhibition of binding by calmodulin and CaMKII; (2) enhanced binding due to dissociation of ICAM-5. The blocking effect is likely more important for mature spines, as calmodulin and CaMKII are known to be enriched in PSD of mature spines; whereas the enhanced binding could occur more often in developing spines, in which ICAM-5 is strongly expressed. Interestingly, we observed a slight decrease in GluN1 and α-actinin binding and shrinkage of the size of spine heads (Tian et al., 2007) in ICAM-5 −/− neurons upon NMDAR activation. In these neurons, spines are relatively more mature, which could generate larger calcium responses, leading to reduced α-actinin/GluN1 binding (Krupp et al., 1999; Leonard et al., 2002; Merrill et al., 2007).

ICAM-5 is also known to colocalize with the ERM protein ezrin in filopodia and this interaction may be important in filopodia formation (Furutani et al., 2007; Furutani et al., 2012). Thus it is plausible to assume that the ICAM-5 interaction with the cytoskeleton in filopodia is complex and involves several components and regulatory elements, which still are incompletely known. Obviously, further detailed studies are therefore needed.

To sum up, we propose a schematic model of ICAM-5-mediated NMDAR activity-dependent spine maturation. In filopodia and immature spines, ICAM-5 is abundant, and binds to β1 integrins in the pre-synaptic terminal. In these structures, α-actinin remains highly dynamic by interacting with the cytoplasmic tail of ICAM-5. Activation of NMDARs leads to shedding of ICAM-5 ectodomain, resulting in dissociation of its cytoplasmic tail from α-actinin, which is then bound to NMDA receptors and becomes clustered at the spine heads. The enrichment of α-actinin contributes to more stable and mature spines by crosslinking the actin filaments and strengthening the actin cytoskeleton.

## MATERIALS AND METHODS

### Reagents and antibodies

Polyclonal antiserum against the cytoplasmic domain of mouse ICAM-5 was provided by Y. Yoshihara. The pAb 1000J recognizing ICAM-5 ectodomains was a gift from P. Kilgannon (ICOS Corporation, Seattle, WA). The following antibodies were purchased: anti-NR1 mAb clone 54.1 (BD Biosciences), anti-α-actinin mAb clone EA-53 (Sigma), anti-α-actinin pAb clone A2543 and anti-α-actinin MAB1682 and a mouse negative IgG (Millipore), peroxidase-conjugated anti-mouse and anti-rabbit pAbs (GE Healthcare), peroxidase-conjugated streptavidin antibody (Thermo Fisher Scientific), peroxidase-conjugated His_6_ antibody (Qiagen) and Cy3-conjugated anti-mouse IgG (Invitrogen). TRITC-conjugated phalloidin for actin staining was purchased from Molecular Probes. The PEF-BOS-ICAM-5 construct was made as described (Tian et al., 1997). The cross-linking reagent dimethyl pimelimidate was purchased from Sigma.

### Animal, cell cultures and transfection

The C57B/l6 mouse strain was used in this study. ICAM-5 −/− mice were generated by gene targeting (Nakamura et al., 2001). All experiments were approved by and performed according to the guidelines of the local animal ethical committee. Hippocampal and cortical neurons were cultured from E18 mouse embryos as described earlier (Nyman-Huttunen et al., 2006).

11 DIV hippocampal neurons were transfected using Lipofectamine 2000 (Life Technologies). For ICAM-5 rescue experiments, plasmid p-EGFP-N1 and ICAM-5 constructs (PEF-BOS-ICAM-5, PEF-BOS-ICAM-5 Δcp or the empty vector) were mixed at 1:10 ratio and co-transfected into neurons. Cells were fixed 24 h after transfection.

Paju cells were cultured in Dulbecco's modifies eagle medium (DMEM, Gibco) with 10% fetal bovine serum, 1% L-glutamine and 1% penicillin-streptomycin at 37°C with 5% CO_2_.

### Recombinant proteins

The His_6_-tagged GluN1 cytoplasmic domain representing residues 834–938 was subcloned into the NdeI/XhoI site of pET21b, and expressed in *E. coli* BL21(DE3)pLysS (Stratagene, La Jolla, CA). All PCR-derived clones were verified by sequencing. The GST-α-actinin and the GST-ICAM-5cyto fusion proteins representing the ICAM-5 cytoplasmic domain were purified by affinity chromatography as described previously (Gilmore et al., 1994; Nyman-Huttunen et al., 2006).

### ELISA

The GST tag was removed by thrombin (GE Healthcare) from GST-ICAM-5 fusion proteins. Ten µg/ml of cleaved ICAM-5 WT, mutated or His_6_-GluN1 cytoplasmic protein were coated on 96-well plates, blocked with 5% BSA (ICAM-5 peptides) or 2% sucrose/0.1% BSA/0.9% NaCl (His_6_-GluN1cyto), and incubated with GST-α-actinin protein R1, R2, R3, R4 or R1–4 or GST for 1 h. Unspecific proteins were removed by washing three times. The amount of bound GST-α-actinin proteins was detected with peroxidase-conjugated GST antibody at 37°C for 1 h. The absorbance at 492 nm was measured.

### Competition assays with GST-α-actinin fusion proteins

Two µg of purified GST-α-actinin R2 fusion proteins or GST were incubated with Glutathione-Sepharose 4B (GE Healthcare) for 1 h at 4°C. After washing, the coupled sepharose was incubated with 10 mM dimethyl pimelimidate for 1 h at room temperature (RT) to secure the binding of GST fusion proteins to the sepharose. One ml of ICAM-5 or His_6_-GluN1 cytodomains (250 nM) was incubated with crosslinked sepharose at RT for 1 h, with increasing amount of purified His_6_-GluN1 or ICAM-5. Proteins bound to sepharose were separated using 4–12% gradient gels (Novex, Invitrogen), and examined by western blotting. Band intensity was quantitated by ImageJ, and data points were fitted to the exponential decay curves using SigmaPlot 11.0.

### Co-immunoprecipitation

Immunoprecipitation was performed as described earlier (Ning et al., 2013). Two hundred µg total protein from P14 WT or ICAM-5 −/− mouse forebrain homogenates or Paju lysates were used for immunoprecipitation. The α-actinin monoclonal antibody (mAb) EA-53 was used to precipitate α-actinin and non-immune IgG was used as a negative control. Bound proteins were detected by western blotting using anti-α-actinin mAb, anti-GluN1 mAb. ICAM-5 was detected using anti-ICAM-5 cytoplasmic domain antiserum, except for Paju cells where an ectodomain recognizing antibody was used. The same experiment was repeated three times.

### Cell stimulation

13 DIV cortical (for immunoprecipitation) or 12 DIV hippocampal (for immunofluorescent staining) neurons were incubated for 1 h at 37°C in Hank's Balanced Saline Solution (HBSS, Gibco) containing 1.8 mM CaCl_2_ without or with 20 µM NMDA. After incubation, cells were washed with PBS, lysed or fixed.

### Immunofluorescence staining

Cells were fixed with PBS containing 4% paraformaldehyde (PFA) and 4% sucrose at 37°C for 15 min and permeabilized with 0.25% Triton X-100 at RT for 5 min. For staining using antibody GluN1 mAb, neurons were fixed with methanol at −20°C for 10 min. Fixed cells were blocked with 5% BSA/PBS at RT for 1 h and incubated with primary antibody overnight at +4°C, followed by 1 h incubation with secondary antibody at RT. Fluorescent images were taken with a confocal microscope (TCS SP5, Leica) using a 63× objective. Within individual experiments, images were acquired using the same channel settings for all samples. Images were processed with Photoshop and ImageJ (National Institutes of Health), and only brightness and contrast were adjusted to remove noise without changing the signals.

### Live-cell imaging

Cultured hippocampal neurons were transfected at 11 DIV with mKATE-α-actinin construct (Evrogen) and monitored at 12 DIV with a high-content confocal microscope (TCS SP5 II, HCSA, Leica) using the 63× objective. Neurons were transferred to HBSS/Ca^++^ medium and monitored for 10 min before NMDA treatment. The same volume of HBSS/Ca^++^ medium containing 40 µM NMDA was added to cultures and incubated for 5 min before restarting imaging. The same neuron was imaged another 20 min. Imaging was done at +37°C in 5% CO_2_.

### Quantitative analysis of immunofluorescent images

For quantitative analysis, images were randomly selected from more than 15 neurons, based on three independent experiments. Brightness and contrast were adjusted, and the background intensity value was subtracted from the image content. The adjustment was aimed to enhance the signal-to-noise ratio, and was applied to all images in a similar way. Segments of dendrites less than 100 µm apart from the somas were used for quantification. Imaging analysis was performed with the same criteria in all experiments and the genotypes or treatments were unknown to the analyzer.

For colocalization analysis, the regions of interest (ROI) were drawn manually in a random manner for selected dendrites or spines. Colocalization between two channels within the ROIs was evaluated by measuring Manders' Coefficient (ImageJ, National Institutes of Health).

For spine analysis, dendritic protrusions were counted as spines when the length is between 1–5 µm with an enlarged head. Spines were selected manually, and the total number was counted in ImageJ.

α-Actinin and actin puncta analysis was performed as described (Glynn and McAllister, 2006). For each dendrite segment, 10 ROIs containing non-specific staining were manually selected from the image as background. The mean value and the standard deviation (s.d.) of fluorescent intensity from these points were calculated. Background intensity value was subtracted at the threshold = Mean+2*s.d. Punctae were selected manually after background subtraction. The number or the total integrated fluorescent intensity of selected punctae was measured by ImageJ.

### Statistical analysis

Data are presented as the mean+s.d. Non-paired t-test was used to measure the inter-group differences between datasets.

### List of abbreviations

ERM, ezrin, radixin and moesin; ICAM, intercellular adhesion molecule; mAb, monoclonal antibody; MMP, matrix metalloproteinase; NMDA, N-methyl-D-aspartic acid; PSD, post-synaptic density; RT, room temperature; sICAM-5, soluble ICAM-5; WT, wild type.

## Supplementary Material

Supplementary Material
