# Supplementary material for: ICAM-5 affects spine maturation by regulation of NMDA receptor binding to α-actinin
Source: Biol Open. 2015 Jan 8;4(2):125–36. doi: 10.1242/bio.201410439 (PMC4365481; doi:10.1242/bio.201410439)
Supplement: Supplementary Material [file supp_4_2_125__index.html]

ICAM-5 affects spine maturation by regulation of NMDA receptor binding to α-actinin — Supplementary Material 

# ICAM-5 affects spine maturation by regulation of NMDA receptor binding to α-actinin

## bio.201410439 Supplementary Material

**Files in this Data Supplement:**

- Supplementary Material - Lin Ning et al. doi: 10.1242/bio.201410439
- Movie 1 - **Movie 1. WT neurons before NMDA treatment.** 11 DIV WT hippocampal neurons were transfected with mKATE-α-actinin and treated with 20 µM NMDA the following day. Dynamics of α-actinin in these neurons was monitored by live cell imaging. This movie was reconstructed from images collected during 10 min before NMDA treatment.
- Movie 2 - **Movie 2. WT neurons after NMDA treatment.** 11 DIV WT hippocampal neurons were transfected with mKATE-α-actinin and treated with 20 µM NMDA the following day. Dynamics of α-actinin in these neurons was monitored by live cell imaging. This movie was reconstructed from images collected during 20 min after NMDA treatment.
- Movie 3 - **Movie 3. ICAM-5 KO neurons before NMDA treatment.** 11 DIV ICAM-5 −/− hippocampal neurons were transfected with mKATE-α-actinin and treated with 20 µM NMDA the following day. Dynamics of α-actinin in these neurons was monitored by live cell imaging. This movie was reconstructed from images collected during 10 min before NMDA treatment.
- Movie 4 - **Movie 4. ICAM-5 KO neurons after NMDA treatment.** 11 DIV ICAM-5 −/− hippocampal neurons were transfected with mKATE-α-actinin and treated with 20 µM NMDA the following day. Dynamics of α-actinin in these neurons was monitored by live cell imaging. This movie was reconstructed from images collected during 20 min after NMDA treatment.
