# Supplementary material for: ICAM-5 affects spine maturation by regulation of NMDA receptor binding to α-actinin
Source: Biol Open. 2015 Jan 8;4(2):125–36. doi: 10.1242/bio.201410439 (PMC4365481; doi:10.1242/bio.201410439)
Supplement: Supplementary Material [file supp_bio.201410439_bio.201410439-s1.pdf]

Supplementary Material
Lin Ning et al. doi: 10.1242/bio.201410439

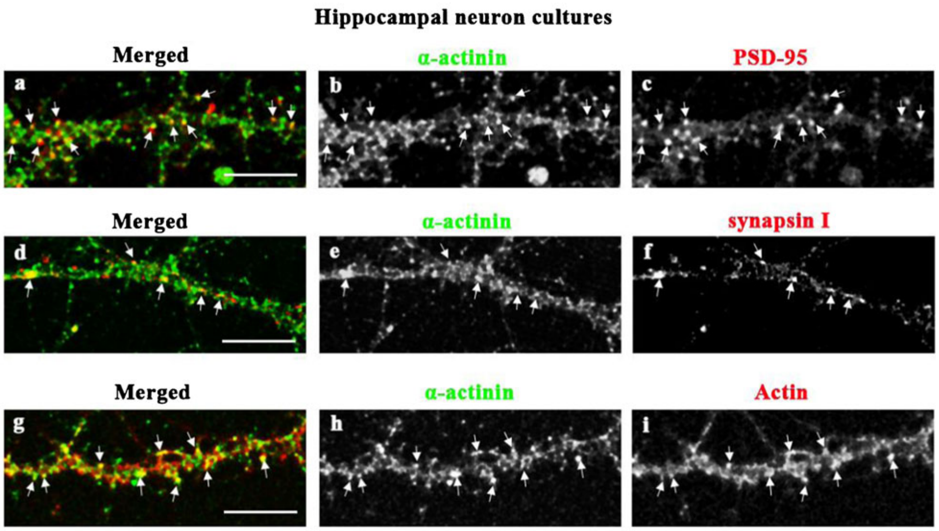

Fig. S1. Colocalization of α-actinin with synaptic markers. 14 DIV hippocampal neurons were fixed with methanol (a–f), or PFA (g–i), and double immunostained for α-actinin and PSD, synapsinI or actin, respectively. α-Actinin exhibits similar pattern at both fixation methods. α-Actinin largely colocalizes with actin. Particularly, α-actinin punctae almost overlapped with the actin-rich area along dendritic shafts, suggesting that α-actinin punctae mostly are located in spines (g). In addition, PSD-95 and synapsin I both colocalize with α-actinin (a and d). Arrows indicate α-actinin punctae colocalized with PSD-95, synapsin I or Actin. Scale bar=20 μm.

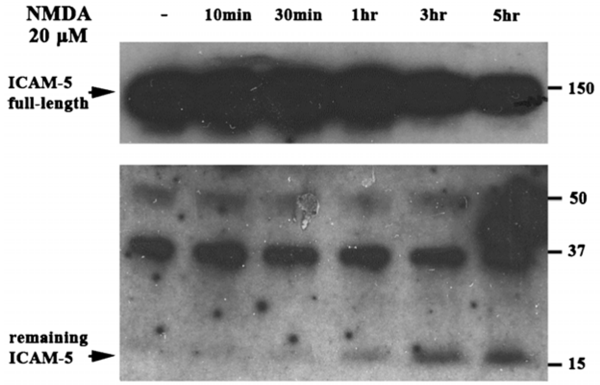

Fig. S2. NMDA induced ICAM-5 cleavage and degradation. 12 DIV hippocampal neurons were treated with 20 μM NMDA for indicated time course. Cells were immediately lysed after the treatment, and 5 μg lysates from each condition were loaded for SDS-PAGE, followed by western blotting. ICAM-5 was examined using an antibody against its cytoplasmic domain. The amount of full-length ICAM-5 (150 kDa) started to decline after 10 min treatment. A 16 kDa band corresponding to the cleaved, membrane-remaining ICAM-5 was barely seen. With prolonged exposure time, the band was weakly detected after 1 h treatment. Upper film was exposed for 1 min; while the lower film was exposed for 1 h.

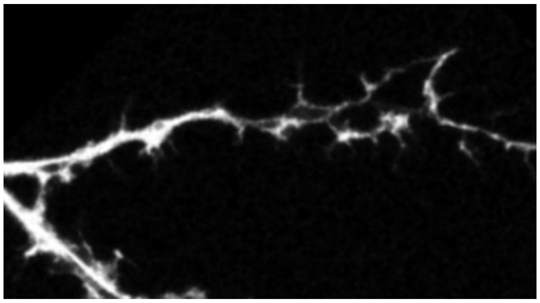

Movie 1. WT neurons before NMDA treatment. 11 DIV WT hippocampal neurons were transfected with mKATE-α-actinin and treated with 20 μM NMDA the following day. Dynamics of α-actinin in these neurons was monitored by live cell imaging. This movie was reconstructed from images collected during 10 min before NMDA treatment.

Biology Open (2015) 000, 1–12 doi:10.1242/bio.201410439

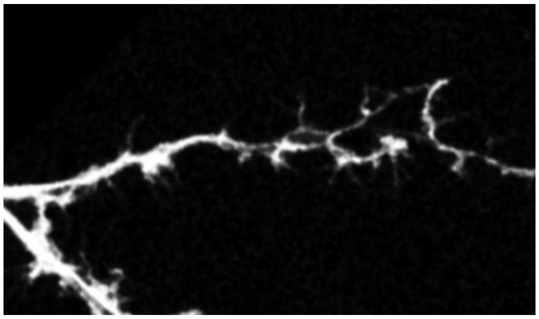

Movie 2. WT neurons after NMDA treatment. 11 DIV WT hippocampal neurons were transfected with mKATE-α-actinin and treated with 20 µM NMDA the following day. Dynamics of α-actinin in these neurons was monitored by live cell imaging. This movie was reconstructed from images collected during 20 min after NMDA treatment.

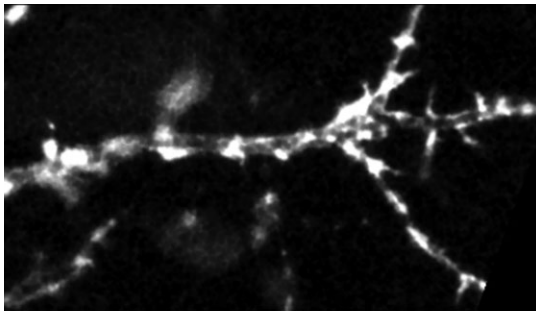

Movie 3. ICAM-5 KO neurons before NMDA treatment. 11 DIV ICAM-5 −/− hippocampal neurons were transfected with mKATE-α-actinin and treated with 20 µM NMDA the following day. Dynamics of α-actinin in these neurons was monitored by live cell imaging. This movie was reconstructed from images collected during 10 min before NMDA treatment.

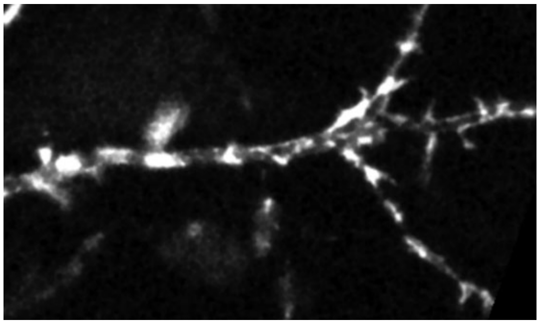

Movie 4. ICAM-5 KO neurons after NMDA treatment. 11 DIV ICAM-5 −/− hippocampal neurons were transfected with mKATE-α-actinin and treated with 20 µM NMDA the following day. Dynamics of α-actinin in these neurons was monitored by live cell imaging. This movie was reconstructed from images collected during 20 min after NMDA treatment.
